# Evaluation of Rheological Properties of Warm Mix Flame-Retardant Asphalt (WMFRA) Binder Suitable for Tunnel Area

**DOI:** 10.3390/polym17212829

**Published:** 2025-10-23

**Authors:** Bo Zhang, Juan Liu, Qiaoli Le, Zhen Lu

**Affiliations:** School of Civil Engineering and Architecture, Guizhou Minzu University, Guiyang 550025, China

**Keywords:** warm mix flame retardant asphalt (WMFRA), rheological properties, flame retardant performance, fatigue property, tunnel area

## Abstract

This study aimed to systematically evaluate the rheological properties of warm mix flame-retardant asphalt (WMFRA). First, conventional performance tests were conducted on the prepared warm mix rubberized asphalt (WMRA), incorporating different warm mix agents in order to screen out an agent with optimum performance. Subsequently, limestone power (LP), aluminum trihydrate (ATH), OA composed of ATH and organically modified montmorillonite (OMMT), and zinc borate (ZK) were employed in the oxygen index (OI) test of WMFRA to determine the optimal dosage of flame retardants. Finally, a dynamic shear rheometer (DSR) and a bending beam rheometer (BBR) were used to evaluate the rheological properties of WMFRA. The results showed that the R-Type warm mix agent was superior to S-Type in reducing consistency and improving low-temperature cracking resistance but slightly weakened high-temperature stability. The OA composite flame retardant could enhance the OI from 20.16% to 24% at 15wt% dosage, thereby meeting the specified flame-retardant requirement. Furthermore, OA could markedly boost the high-temperature performance of WMFRA, exhibiting significantly higher complex modulus (G*) and rutting factor (G*/sinδ) compared to WMFRA with other flame retardants. In general, all flame retardants reduced the temperature sensitivity of WMFRA, with ZK being the most effective at 12.6%. Regarding low-temperature performance, LP and ATH improved stress relaxation of WMFRA, while ZK and OA impaired this capability. All flame retardants reduced low-temperature flexibility, but the low-temperature behavior was still dominated by the S(t). For fatigue performance, LP and ATH degraded the fatigue performance by advancing the damage time by 958.9 s and 669.7 s, respectively. In contrast, ZK improved fatigue performance by increasing the complex shear modulus, thereby extending the fatigue life (*N_f50_*) by 3.2%. This study provided a theoretical basis for the formulation optimization of WMFRA.

## 1. Introduction

Asphalt pavements were the cornerstones of modern transportation infrastructure, whose performance was directly related to the safety, durability, and comfort of roads [1]. In specific engineering environments, such as highway tunnels, more stringent and specific requirements were posed on pavement materials [2]. Because tunnels are enclosed areas, heat and smoke cannot escape rapidly if a fire starts, increasing the likelihood of serious safety incidents. Therefore, asphalt pavements applied in tunnels not only needed to possess conventional pavement performance but also had to have excellent flame-retardant performance, so as to delay the spread of fires, reduce the release of toxic smoke, and gain valuable time for personnel evacuation and fire rescue [3,4].

To meet the flame-retardant requirements of tunnel pavements, flame-retardant asphalt (FRA) technology emerged. In the beginning, flame retardant materials were used in construction, textile, electronics, and aerospace applications, such as nanomaterials like tannic acid (TA), phytic acid (PA), lignin, deep eutectic solvents (DES), and metal–organic skeleton materials (MOFs), with promising results [5,6]. Afterwards, the technology was cited in the field of asphalt material for the preparation of FRA. Commonly used asphalt flame retardants included inorganic agents, organic agents, reactive agents, and expansion agents. These agents exerted flame-retardant effects through multiple mechanisms, such as undergoing endothermic decomposition during combustion, forming insulating char layers, diluting the concentrations of combustible gases and oxygen, and interrupting free radical chain reactions [7,8]. Currently, some scholars have already studied the improvement of the suitability of asphalt in tunnel pavements by adding flame retardants to asphalt. Liu et al. [9] modified asphalt with environmentally friendly flame retardants, including aluminum trihydrate (ATH), montmorillonite (MMT), and talc (LDHs), and compounded FRA with polyurethane (PU). The results showed that the compounding of PU with flame retardants could notably improve the high- and low-temperature performance and flame-retardant properties of asphalt. Among the three flame retardants, LDHs showed the best flame-retardant effect. Tian et al. [10] aimed at the problem of limited use caused by the high cost of traditional halogens, antimony, boron, and other flame retardants, choosing three cheap phosphorus nitrogen flame retardants and two inorganic flame retardants as the objects, and analyzed the influence of single-component and multi-component flame retardants on the flame retardant performance of asphalt based on the limiting oxygen index (OI). It was found that the phosphorus–aluminum composite system exhibited good flame-retardant performance. Its flame-retardant performance reached the Class B flame-retardant standard, which could meet the application requirements for tunnel surface layers, and also greatly reduced the techno-economic costs of flame-retardant asphalt. Li et al. [11] prepared and evaluated a new composite FRA through a series of tests based on four components of asphalt. The results showed the saturated fraction and aromatic fraction of FRA had poor thermal stability, while the resin and asphaltene performed better in combustion. The increase in the amount of flame retardant made the asphalt softening point difference increase, and the flame retardant was occasionally agglomerated but existed mostly in single particles with good dispersion. Tan et al. [12] prepared the nanocomposite flame retardant (NCFR) composed of halloysite nanotubes (HNTs) and organically modified montmorillonite (OMMT) for studying rheological and flame-retardant properties of FRA. It can be concluded that HNTs could significantly increase the OI and auto-ignition temperature of FRA and boost its high- and low-temperature properties as well as the integrity of the barrier layer, whereas OMMT could strongly suppress smoke generation and improve the high-temperature performance, but it would deteriorate the low-temperature performance. In summary, most flame retardants, although having good flame-retardant properties, may cause a decrease in the high-temperature stability or low-temperature cracking resistance of FRA. Therefore, while pursuing flame retardant efficacy, it was important to systematically evaluate its impact on the rheological properties of asphalt. Considering that flame retardants such as alumina trihydrate (ATH), zinc borate (ZK), and OMMT were non-toxic compared to halogenated flame retardants, and that phosphorus flame retardants were not sufficiently resistant to migration, these three flame retardants met the current technological demand for flame-retardant materials with high efficiency [13], low toxicity [14], and multi-functionality [15], and had become the target of this study on flame retardants.

On the other hand, with an increasing number of global emphases on environmental protection and sustainable development, warm mix asphalt (WMA) technology had been widely favored for its ability to significantly reduce the production and paving temperatures of asphalt mixtures, typically by 20–40 °C [16]. WMA technology reduced the viscosity of the asphalt through the addition of warm mixing agents such as organic waxes [17], surfactants [18], synthetic zeolites [19], or nanomaterials [20] to enable low-temperature construction. Several studies have been conducted on warm mix asphalt. Han et al. [21] designed functional waxes containing oxygen groups, amide groups, and long-chain aliphatic hydrocarbons and mixed them with asphalt melt. The results showed that the long-chain aliphatic hydrocarbon warm mix agents significantly improved the high-temperature rheology of asphalt. Oxygenated groups improved the low-temperature cracking resistance. Amide groups improved the surface roughness. Gao et al. [22] investigated the influence of warm mix agent on the rheological properties of SBS warm mix modified asphalt and its viscosity-reducing mechanism. The results showed that after the addition of warm mix agent, the viscosity of the SBS-WMA decreased and its high-temperature performance improved. Dong et al. [23] prepared foamed WMA with different foaming water contents. After being treated with RTFOT (Rotating Thin Film Oven Test) and PAV (Pressure Aging Vessel Test), they tested its conventional properties, rheological properties, and fatigue resistance via conventional tests and DSR tests. The conclusions showed that the foamed WMA exhibited better high-temperature performance but poorer fatigue resistance than that of foamless asphalt. Huang et al. [24] studied warm mix crumb rubber asphalt (WMRA) by constructing its molecular models using Materials Studio software (MS 2023). The results showed that the warm mix agent altered the properties of WMRA, such as glass transition temperature and surface morphology roughness. However, S-type warm mix agent and R-type warm mix agent are widely used due to their advantages of easy availability of raw materials, mature production technology, low construction cost, and excellent performance. S-type warm mix agent was to reduce the viscosity of asphalt through physical action, focusing on high-temperature performance optimization, which improved the high-temperature stability and water stability of asphalt, especially suitable for hot and rainy areas. Chen et al. [25] investigated the effects of warm mix agents (3% foamed warm mix, 3% Sasobit, and 0.8% Evotherm) on the physical and rheological properties of High Viscosity Modified Asphalt (HVMA). They found that all four technologies could reduce the construction temperature and improve the high-temperature rheological properties but had adverse effects on the low-temperature cracking resistance of the asphalt. Tong et al. [26] stated that Sasobit warm mix agent can reduce the needle penetration and ductility of asphalt and increase the softening point. Its viscosity-reducing effect was mainly realized by adsorption of saturated components and lowering the bond energy between 1H and other molecules. R-type warm mix agent was based on the surfactant chemistry of the warm mix technology, which was characterized by the molecular level of interfacial regulation to achieve efficient viscosity reduction and performance optimization. Compared with S-type warm mix agent, R-type warm mix agent had better low-temperature performance and weakened high-temperature performance but still met the specification requirements. Zhang et al. [27] conducted a series of studies by blending Rediset warm mix agent into asphalt and found that Rediset could increase the penetration and softening point of asphalt and make asphalt harder. Thus, S-type warm mix agent and R-type warm mix agent as a typical representative of the two viscosity reduction mechanisms of physical viscosity reduction and chemical interface regulation, and both had the advantage of high maturity in engineering applications, which can effectively avoid the limitations of other single-type warm mix agents in terms of technical representativeness or engineering practicability.

The technology of warm mix flame-retardant asphalt (WMFRA) binders by combining WMA with FRA was a very promising development direction in the field of tunnel asphalt pavements. However, the current research on WMFRA mostly remains in the preliminary exploration of single flame-retardant efficiency or construction temperature, and there is a serious lack of systematic evaluation of its rheological properties. Therefore, it is urgent to conduct in-depth research on the rheological behavior of WMFRA in order to ensure the long-term rutting and cracking resistance of tunnel pavements.

**Figure 1:**
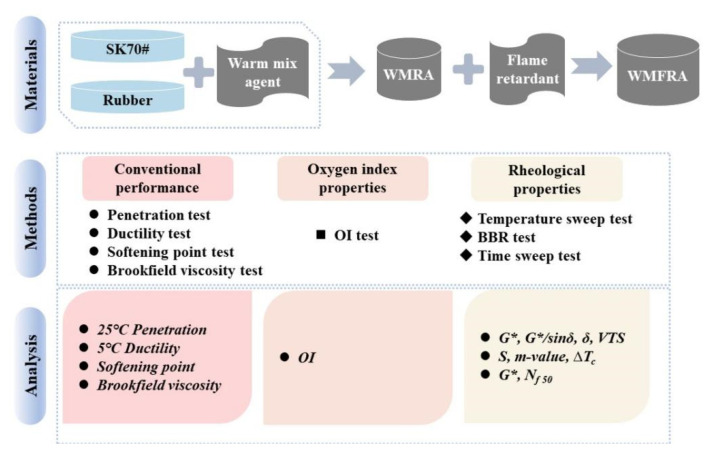
The flowchart.

Therefore, in order to systematically evaluate the rheological properties of warm mix flame-retardant asphalt (WMFRA) in the tunnel area, the warm mix agent used and its dosage were firstly determined by conventional performance tests. Subsequently, WMFRA binders were prepared and the dosage of flame retardant was determined by the oxygen index (OI) test. Based on this, the rheological properties of WMFRA were finally investigated by the dynamic shear rheometer (DSR) and bending beam rheometer (BBR). This detailed flowchart is illustrated in Figure 1.

## 2. Materials and Methods

### 2.1. Raw Materials

The matrix asphalt selected for this study was SK70#, produced in Guiyang, China, whose basic performance indexes are shown in Table 1.

The waste tire produced in Guiyang, China, was used as raw material to produce 60 mesh rubber powder by crushing and grinding at room temperature.

S-type warm mix agent is a viscosity-reducing agent with low-melting-point organic compounds as the core, which is produced in Shanghai, China. And its mechanism of action is to melt into liquid state at a high temperature to reduce the viscosity of asphalt at a high temperature, so as to realize the low-temperature construction. R-type warm mix agent is manufactured in Chengdu, China, with surfactants as the core ingredient. It is used to reduce the surface tension between asphalt and aggregates to improve the asphalt for the aggregates of the cohesive properties of the workability of the mixture. Their physical properties are displayed in Table 2 and Table 3, respectively.

The flame retardants used in this study included limestone power (LP), ATH, ZK, and composite OMMT and ATH (OA). LP and ATH are produced in Guiyang, China. ZK and OMMT are produced in Zhangjiagang, China and Hangzhou, China, respectively. In order to study the actual effects of different flame retardants on the rheological properties of WMFRA, and to make the performance test of WMFRA more accurately respond to the impact of flame retardants on the actual properties of the mixture, the RA mortar was prepared by using LP for comparative study with other WMFRA.

OMMT could be detrimental to the low-temperature performance of asphalt; therefore, the dosage of OMMT was fixed at 3.5% of the mass of rubber asphalt (RA), and the dosages of ATH were 3%, 6%, 9%, 12%, and 15%, respectively. In addition, the technical indicators of ATH, ZK, OMMT, and LP are shown in Table 4, Table 5, Table 6 and Table 7.

The flowchart is displayed in Figure 1.

### 2.2. Sample Preparation

First, RA was prepared using a high-speed shear. The matrix asphalt was heated to a constant temperature of 150 °C to a flowing state, and 16% of the rubber powder was added and stirred with a glass rod to make the rubber powder disperse in the matrix asphalt. The high-speed shear was turned on, and the shear speed was increased to 5000 r/min; the shear time and temperature were controlled at 35 min and 170 °C, respectively.

Next, referring to the findings of relevant scholars [28,29], it was found that the dosage of warm mix agent does not need to be too high to make a significant impact on asphalt properties. Thus, the S-type warm mix agent (2%, 3%, and 4% of the mass of RA) was added to the developed RA and stirred for 15 min to produce the S-type warm mix rubber asphalt (S-WMRA). Similarly, the R-type warm mix agent (0.6%, 1.2%, and 1.8% of the mass of RA) was added to the developed RA by dropping it into a burette and mixing for 15 min to produce the R-type warm mix rubber asphalt (R-WMRA).

Ultimately, different flame retardants (LP, ATH, ZK, and OA) were added to the WMRA to prepare the WMFRA.

### 2.3. Characterization

#### 2.3.1. Conventional Performance Test

Conventional performance tests were conducted on WMRA with different dosages to test the penetration, ductility, softening point, and Brookfield viscosity to evaluate the effect of different warm mix agents on the conventional performance of WMRA.

#### 2.3.2. High Temperature Rheological Test

Asphalt binders were subjected to temperature sweep tests utilizing a dynamic shear rheometer (TA, DHR-1, produced in Burladingen, Germany). The tests were conducted using parallel plates that were 25 mm in diameter and 1 mm thick. The temperature range was 52 °C to 88 °C at intervals of 6 °C, and the angular frequency was set to 10 rad/s. The strain was set to 1.25% while using the strain control mode. Studies have shown that the viscosity temperature sensitivity (VTS) accurately reflects the temperature sensitivity of asphalt binders compared to the penetration index derived from the penetration test. By plotting the relationship between logT and $log\eta^{\prime }$ and performing a linear regression, the absolute value of the slope of the regression line is the VTS, and the calculations are shown in Equations (1) and (2).

(1) $$\eta^{\prime }=\frac{{\left(sin\delta \right)}^{-4.8628|G^{*}|}}{\omega }$$

(2) $$VTS=\frac{\mathrm{lg}\left(lg\eta_{2}\right)-g(lg\eta_{1})}{lgT_{k2}-lgT_{k1}}$$

where $\eta^{\prime },\eta_{1},\eta_{2}$ are viscosities, Pa·s; δ is phase angle, °; $G^{*}$ is complex module, kPa; ω is angular frequency, rad/s; and $T_{k2}$, $T_{k1}$ are Kelvin temperatures, K.

**Figure 2:**
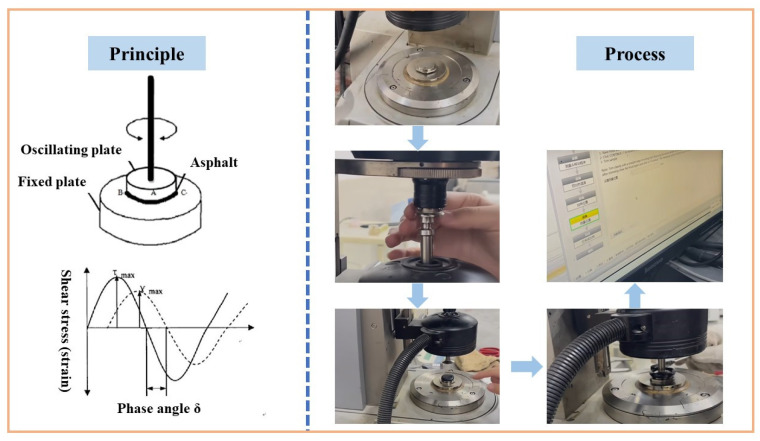
The schematic diagram of the temperature rheological test.

The specific test procedure is shown in Figure 2.

#### 2.3.3. Low Temperature Rheological Test

A bending beam rheometer (BBR) test was used to assess the low-temperature rheological characteristics of WMFRA at −12 °C, −18 °C, and −24 °C. BBR was produced in Graz, Austria. Creep rate (m-value) and stiffness (S) were measured. The $∆T_{C}$ value was defined as the temperature differential between the m-value threshold (corresponding to a temperature with an m-value of 0.3) and the critical temperature based on the stiffness threshold (corresponding to a temperature of 300 MPa stiffness). The equations are displayed in (3)–(5).

(3) $$T_{C,S}=T_{1}+\frac{{(T}_{1}-T_{2})(lg300-lgS_{1})}{lgS_{1}-lgS_{2}}-10$$

(4) $$T_{C,m}=T_{1}+\frac{\left(T_{1}-T_{2})(0.3-m_{1}\right)}{m_{1}-m_{2}}-10$$

(5) $$∆T_{c}={∆T}_{C,S}-{∆T}_{C,m}$$

where $T_{1}$ and $T_{2}$ refer to higher and lower test temperatures; $S_{1}$ and $S_{2}$ refer to S at higher and lower temperatures; and $m_{1}$ and $m_{2}$ refer to m values at higher and lower temperatures, respectively.

#### 2.3.4. Fatigue Test

DSR was employed to conduct a time sweep test for evaluating the fatigue performance of WMFRA. The test was performed with an applied strain of 8%, using 8 mm parallel plates as fixtures, at a test temperature of 25 °C and a loading frequency of 10 Hz.

#### 2.3.5. Oxygen Index Test

A 150 mm × 150 mm × 3 mm metal mold frame, three pieces of glass fiber surfacing mats, and a smooth tile base were prepared. Asphalt was heated at 175 °C for 2 h until molten. After weighing, the mats, together with the mold and tile base, were placed in an oven at 120 ± 2 °C for 40 min. The mold, after being coated with a release agent, was placed on the base, with the mats laid flat and pressed down. The well-stirred asphalt was poured in; after cooling, the mold with the base was frozen for 1 h. The asphalt was then cut into specimens of 110–120 mm in length and (6.5 ± 0.5) mm in width and dried at 35 ± 2 °C for 5 h.

A specimen was mounted on the fixture, with its bottom wrapped in kraft paper, and ignited in air to determine the initial oxygen concentration: 18% for rapidly burning, 21% for slowly burning, and 25% for non-burning. A new specimen was installed in the combustion cylinder, and tests were conducted at 1% oxygen concentration intervals (marked as X if burning lasted over 180 s or exceeded 50 mm in length, otherwise O). The oxygen concentration corresponding to O was taken as the initial concentration when the difference between two test concentrations was ≤1% with different marks. After re-testing the mark at the initial concentration, the oxygen concentration was adjusted in 0.2% increments until the mark reversed, followed by four additional tests. The OI was finally calculated to verify its validity.

## 3. Results and Discussion

### 3.1. Conventional Properties

**Figure 3:**
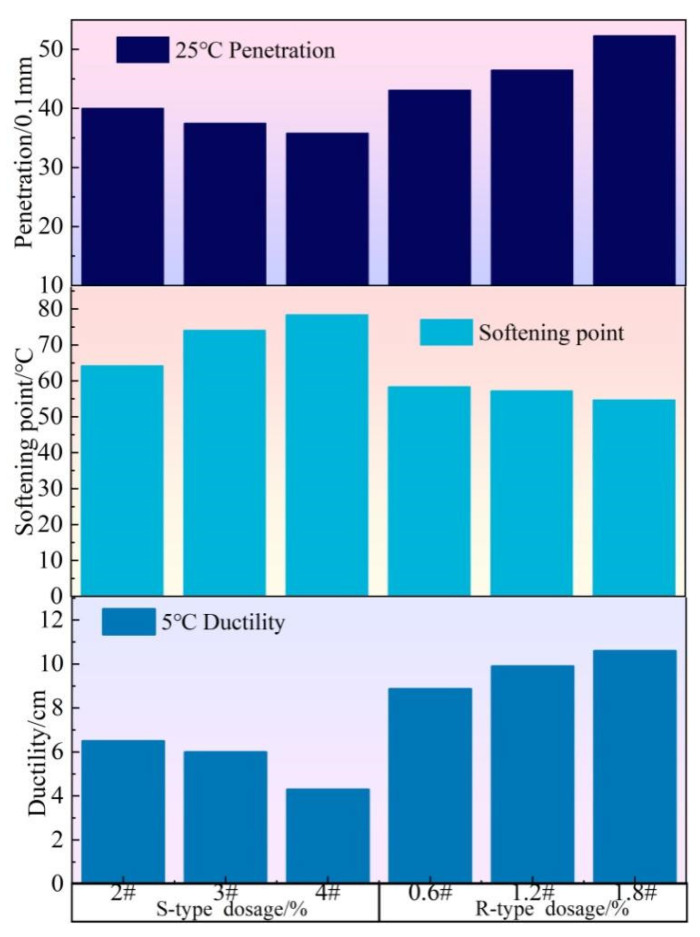
Conventional performance of WMRA.

First, conventional performance tests were conducted on WMRA, and the results are exhibited in Figure 3.

As shown in Figure 3, the penetration of S-type WMRA and R-type WMRA exhibited an entirely opposite trend with the variation in warm mix agent dosage. With the increase in the dosage of S-type warm mix agent, the penetration of WMRA decreased gradually, but the extent of the decrease was relatively low. This indicated that the S-type warm mix agent slightly increased the consistency of WMRA, thereby reducing its fluidity. In contrast, after the incorporation of R-type warm mix agent, the penetration of WMRA increased, and the extent of the increase was relatively significant, demonstrating that the R-type warm mix agent had advantages in reducing the consistency of WMRA and enhancing its fluidity.

The results of softening point and ductility tests showed that S-type warm mix agent can significantly enhance the high-temperature stability of WMRA, but would weaken its low-temperature ductility, and the weakening effect of low-temperature cracking resistance at 3wt% dosage was relatively low. As the dosage of R-type warm mix agent increased, the softening point of WMRA gradually decreased, indicating the drop of high-temperature stability. However, its ductility was rising, indicating the enhancement of low-temperature crack resistance. After comprehensively considering the effect of warm mix agent on the high- and low-temperature performance of WMRA, the dosage of S-type warm mix agent was determined to be 3wt%. R-type warm mix agent could improve the low-temperature performance of WMRA but slightly deteriorate its high-temperature performance. After comprehensively considering the effect of this agent on the high- and low-temperature performance of WMRA, the dosage of R-type warm mix agent was decided to be 1.2wt%.

**Figure 4:**
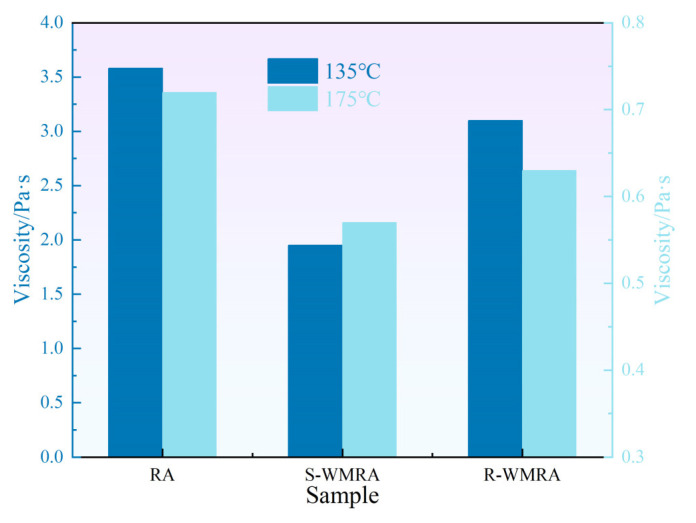
The result of the Brookfield test.

The rubber asphalt (RA), 3wt% dosed S-WMRA, and 1.2wt% dosed R-WMRA were selected for Brookfield viscosity test at 135 °C and 175 °C, and the result is illustrated in Figure 4.

From Figure 4, it can be seen that the viscosity of RA at 135 °C and 175 °C was the largest, followed by R-WMRA, and the viscosity of S-WMRA was the lowest, which suggested that the fluidity of S-type WMRA was the most excellent, and that of RA was the most unfavorable. When comparing RA at 135 °C, the viscosity of S-WMRA and R-WMRA decreased significantly by 46.7% and 15.1%, respectively, and this decrease was more significant compared to that at 175 °C, indicating that the viscosity reduction effect of these two warm mix agents was significant at 135 °C and that the increase in temperature would weaken the viscosity-reducing effect of the warm mix agents. In addition, at any temperature, the viscosity-reducing effect of S-type warm mix agent on RA was significantly greater than that of R-type warm mix agent.

Analyzing the reasons for the viscosity reduction effect of these two warm mix agents on RA, S-type is a viscosity reduction warm mixing agent, which can be mixed into asphalt to regulate the components of asphalt, thus reducing the viscosity of asphalt, improving the fluidity of asphalt, and ultimately achieving the effect of warm mixing. In contrast, R-type is a surface-active agent, which cannot reduce viscosity to achieve the warm mix effect. The surfactant molecules in this type of warm mix agent could act on the micro-interface between mineral aggregates and asphalt, adsorb onto the solid surface, and form an adsorption layer with directional arrangement. This process reduced the interfacial force between asphalt and aggregates, improved the ability of asphalt to wet and coat aggregates, and enhanced the workability of asphalt mixture during mixing and compaction, thus realizing the warm-mixing effect [30,31,32].

### 3.2. Oxygen Index Properties

**Figure 5:**
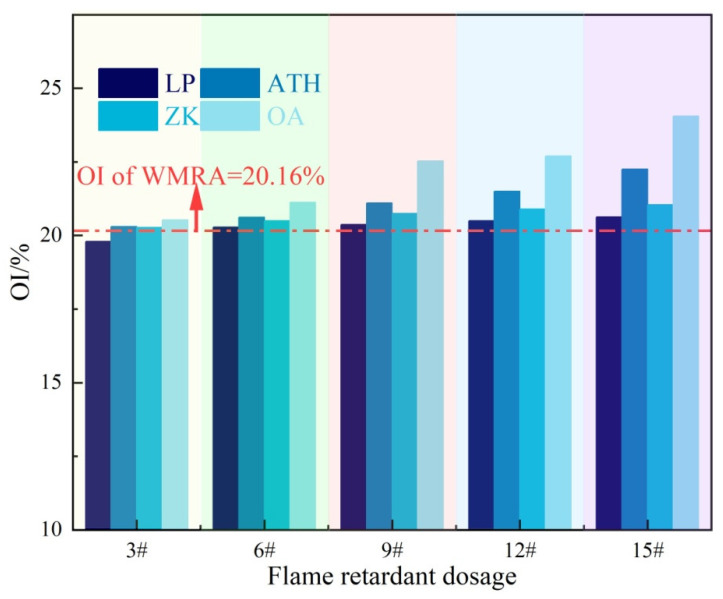
The results of the OI.

Based on the conventional performance of WMRA, R-type warm mix (with a dosage of 1.2%) was selected for follow-up study, and WMFRA was prepared by mixing different flame retardants and conducting oxygen index test. The results are shown in Figure 5.

In Figure 5, the oxygen index (OI) of WMRA is 20.16%, which is much lower than the oxygen concentration of 21% in air. This indicated that WMRA was a flammable material and could continue to burn in air under the action of an external fire source. For LP, at low dosages, LP exerted a slight influence on the OI of WMFRA. However, as its dosage continued to increase to 15%, the OI of WMFRA increased by 47%, and its flame-retardant performance was well-improved. Figure 4 also illustrates that an increase in the dosage of ZK flame retardant could increase the OI of WMFRA, indicating that ZK could improve the flame-retardant performance of WMFRA, and the greater the dosage, the more significant the improvement effect. The flame-retardant effect of ZK was superior to that of LP. Taking a dosage of 15wt% as an example, LP increased the OI of WMFRA to 20.6% at this dosage, while ZK raised this index to 21%. Although it showed an improvement compared with LP, the flame-retardant efficiency of ZK remained relatively low. Even when the dosage reached 15wt%, the OI of WMFRA was still significantly lower than the required 23.0%, failing to meet the application requirements. This indicated that the influence of flame-retardant materials on the flame-retardant performance of WMFRA after incorporation mainly depended on their own properties and was less affected by the reduction in the relative content of asphalt.

The OI of WMFRA increased significantly with the addition of ATH dosage. At a dosage of 3wt%, its improving effect on the OI of WMFRA had already exceeded that of LP and ZK. When the ATH dosage was further increased to 15 wt%, the OI of WMFRA reached 22.25%. This indicated that ATH could markedly boost the flame-retardant performance of WMFRA, and its flame-retardant efficiency was significantly higher than that of LP and ZK. The reason was that when a specific temperature was reached, ATH would decompose and release a large amount of crystalline water. The crystalline water could absorb heat, slow down the heating rate of WMFRA, and thus delay its combustion process. Meanwhile, after the crystalline water was converted into water vapor, it could dilute the combustible gases released by asphalt decomposition, reduce the concentration of combustible gases, and weaken the intensity of combustion [33,34]. However, the flame-retardant mechanism of ATH determined that its flame-retardant efficiency was limited, and a relatively high dosage was required to give full play to its flame-retardant effect. Therefore, it needed to be compounded to enhance its flame-retardant efficiency through the synergistic flame-retardant effect.

The dosage of OMMT in OA was fixed at 3.5wt%. The OI of WMFRA showed a significant increasing trend with the increase in the dosage of ATH in OA. At a content of 9% (i.e., 3.5wt% OMMT + 9wt% ATH), OA could significantly increase the OI of WMFRA to 22.5%, and it exhibited higher flame-retardant efficiency compared with other flame retardants. When the dosage of the OA reached 15% (i.e., 3.5wt% OMMT + 15wt% ATH), the OI of WMFRA was 24%, which was much higher than the required value of 23.0%, meeting the application requirements for flame-retardant asphalt. This could be attributed to the respective flame-retardant mechanisms of ATH and OMMT, as well as the synergistic flame-retardant effect when they were used together. OMMT could be uniformly dispersed in WMFRA, forming intercalated or exfoliated spatial structures, which prevented the contact between oxygen and asphalt. ATH, on the other hand, mainly relied on endothermic decomposition to release water to exert its flame-retardant effect [35,36]. The two could work in tandem since their primary flame-retardant systems differed.

From the OI test results, most WMFRAs showed good flame-retardant performance at a dosage of 15wt%. Accordingly, a 15wt% flame retardant dosage was employed for the subsequent tests on the rheological properties of WMFRA.

### 3.3. High Temperature Rheological Properties

**Figure 6:**
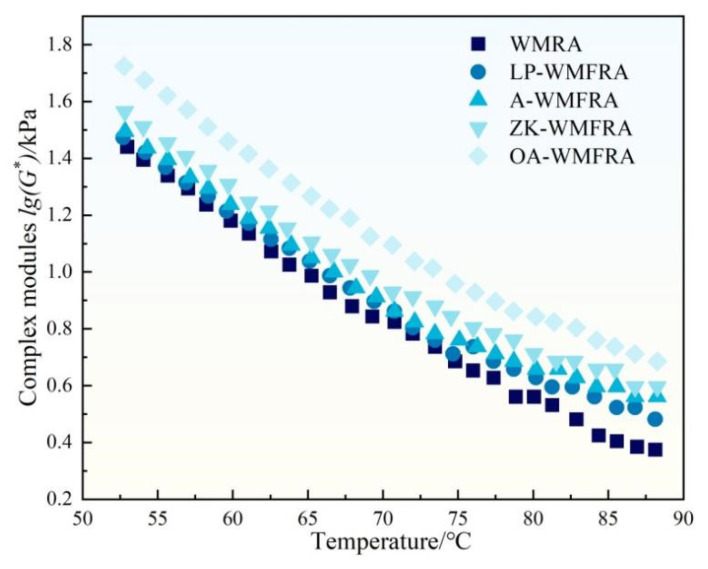
The complex modules of WMFRA in temperature sweep test.

**Figure 7:**
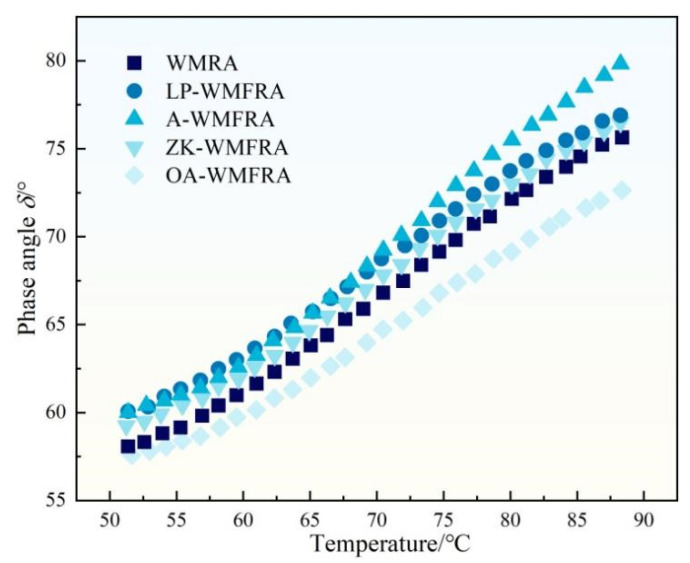
The results of phase angle for WMFRA.

**Figure 8:**
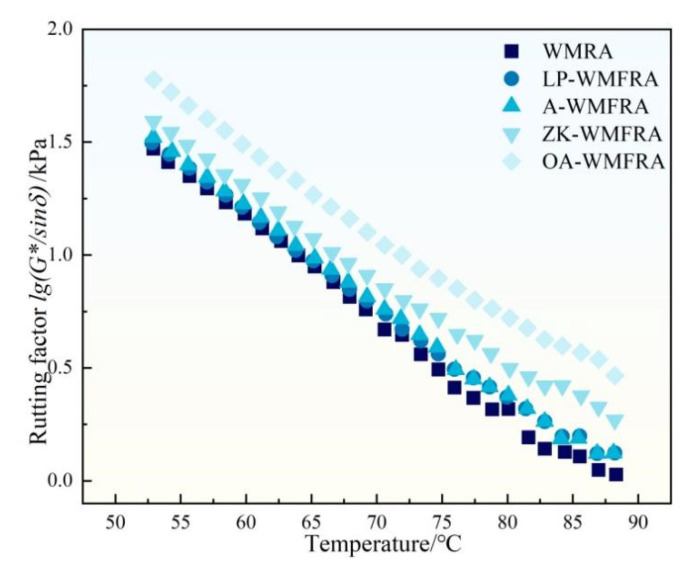
The results of rutting factor for WMFRA.

**Figure 9:**
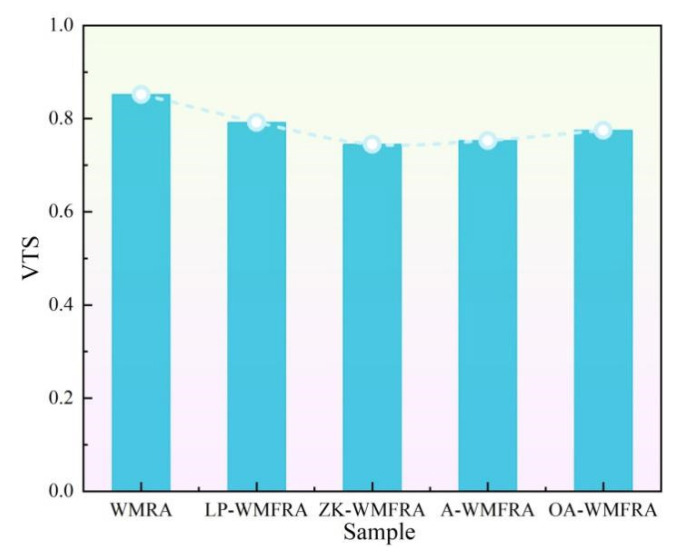
The results of VTS for WMFRA.

The temperature sweep tests of WMFRA were shown in Figure 6, Figure 7, Figure 8 and Figure 9. As depicted in Figure 6, the temperature increase resulted in a decrease in the complex modulus (G*). In comparison to WMRA, the G* of WMFRA increased to different degrees with the addition of different flame retardants. It was noticeable that the G* of OA-WMFRA was higher than that of other WMFRAs, followed by ZK-WMFRA, and the G* of LP-WMFRA was only slightly above that of WMRA, which suggested that the effect of OA flame retardants on the increase of G* was the most significant for WMFRA, followed by ZK; the influence of LP made the smallest effect on the G*. The higher complex modulus indicated the higher high-temperature deformation resistance of asphalt [37,38]. Therefore, the incorporation of flame retardant was beneficial to the improvement of high-temperature deformation resistance of WMFRA, and OA-WMFRA exhibited the strongest high-temperature deformation resistance, followed by ZK-WMFRA and A-WMFRA.

As seen in Figure 7, the phase angle (δ) ordering of different WMFRAs exhibited a pattern different from that of the complex modulus. A larger phase angle signifies a smaller elastic proportion and a larger viscous proportion [39,40]. In addition to OA, ZK, ATH, and LP were incorporated into WMFRA to increase its phase angle, indicating that these flame retardants could enlarge the viscous component and decrease the elastic component of WMFRA. On the contrary, the phase angle of OA-WMFRA was lower compared to WMRA, which suggested that OMMT in the compounded OA flame retardant significantly increased the elastic proportion in WMFRA, which was related to the insertion or exfoliation spatial structure formed by OMMT in the asphalt.

From Figure 8, it can be seen that the rutting factor (G*/sinδ) of WMFRA had been improved to different degrees after mixing different flame retardants, which meant that the high-temperature rutting resistance of WMFRA had been enhanced. This may be due to the fact that the flame-retardant powder did not possess temperature sensitivity. WMFRA gradually softened while the powder material did not produce significant changes when the temperature increased; the flame retardants powders were distributed in WMFRA, which hindered the deformation of WMFRA at high temperatures. Among these flame retardants, LP and ATH did not improve the G*/sinδ significantly. The G*/sinδ of WMRA at 60 °C increased by 3.8% and 6.7% after 15wt% LP and ATH were incorporated, and the improvement effect was not significant. The ZK flame retardant improved the G*/sinδ of WMFRA better than that of LP and ATH at the same dosage level, and it could improve the G*/sinδ of WMFRA at 60 °C by 25.1% at the same dosage level. In the test temperature range, the G*/sinδ of OA-WMFRA was consistently and significantly larger than the other samples, and its G*/sinδ at 60 °C was nearly twice as high as that of WMRA. This was attributed to the fact that OMMT in OA, when uniformly dispersed in asphalt, could form intercalated or exfoliated structures. These structures increased the resistance to the movement of asphalt molecules, hindered the free movement of asphalt molecular chains, inhibited the deformation of asphalt at high temperatures, and thereby improved the high-temperature performance of WMFRA [41]. Comparisons with LP-WMFRA and A-WMFRA showed that ATH in the OA flame retardant had almost no effect on the high-temperature performance of WMFRA, whereas OMMT played a major role in improving the high-temperature performance of WMFRA.

A smaller VTS indicated that the asphalt material was less sensitive to changes in temperature [42,43]. It can be observed from Figure 9 that compared to WMRA, the VTS of WMFRA blended with flame retardant showed a decreasing trend, indicating that the flame retardant can reduce the temperature sensitivity of asphalt, and the ZK flame retardant was the most effective in reducing the temperature sensitivity of WMFRA, which was reduced by 12.6%. The reason was that the flame retardants stabilized the structure of WMFRA through physical filling and polar adsorption and formed a three-dimensional network with asphalt via chemical cross-linking, supplemented by carbon layer assistance, thereby reducing the temperature sensitivity of WMFRA.

### 3.4. Low Temperature Rheological Properties

**Figure 10:**
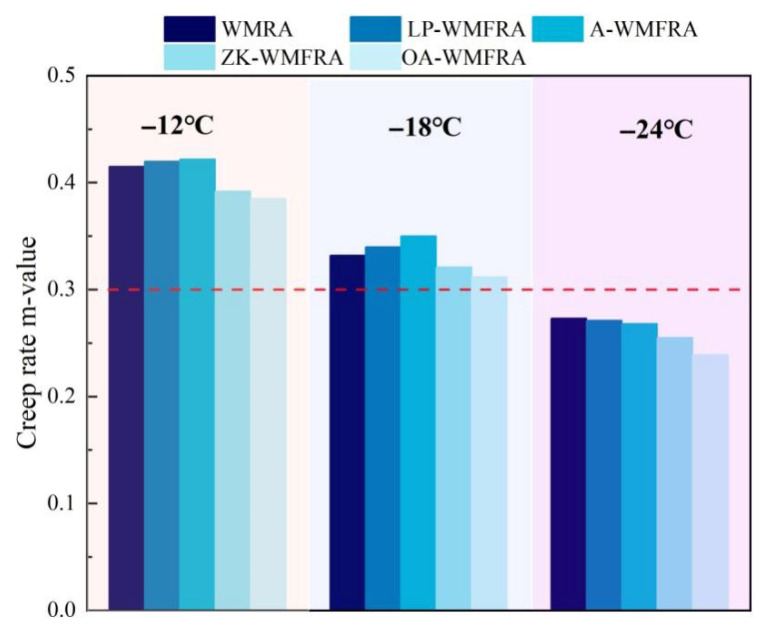
The results of m-value for WMFRA.

As displayed in Figure 10, it was common sense that a significant decrease in temperature led to a dramatic reduction in the m-value. At −12 °C and −18 °C, the m-values of LP-WMFRA and A-WMFRA were higher than those of WMRA, while the m-values of ZK-WMFRA and OA-WMFRA were lower than those of WMRA. This indicated that LP and ATH could significantly improve the stress relaxation capacity of WMFRA, enabling it to better relieve its own stress through deformation. The reason for this was that LP weakened the rigid aggregation of asphalt molecular chains through the formation of hydrogen or chemical bonds between the surface polar groups and the polar components of the asphalt, allowing the molecules to slide easily at low temperatures to release stress. ATH formed polar adsorption with polar components of asphalt through surface hydroxyl groups, which not only hindered the excessive rigid contraction of molecular chains at low temperatures but also buffered the stress transfer with particle dispersion and thus improved the low-temperature stress relaxation ability of asphalt [44]. In contrast, ZK and OA could slightly impair the stress relaxation capacity of WMFRA. Comparing the m-values of A-WMFRA and OA-WMFRA, it was found that the m-value of OA-WMFRA incorporated with OMMT dropped remarkably, which confirmed that OMMT would impair the low-temperature cracking resistance of asphalt. The reason was that OMMT could form intercalated structures and exfoliated structures after being incorporated into asphalt, which could restrain the deformation of asphalt to a certain extent, making it difficult for asphalt to dissipate internal stress through deformation. Macroscopically, this manifested as a decrease in the m-value of asphalt. Although OMMT impaired the low-temperature performance of WMFRA, the m-value of OA-WMFRA met the specification requirements at −18 °C. However, when the temperature dropped to −24 °C, the m-value of each asphalt no longer fulfilled the specification.

**Figure 11:**
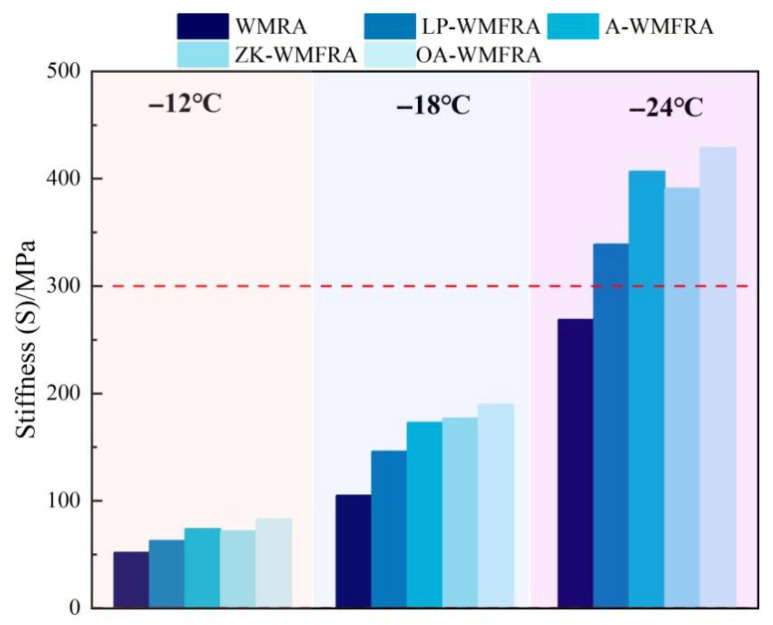
The results of stiffness for WMFRA.

**Figure 12:**
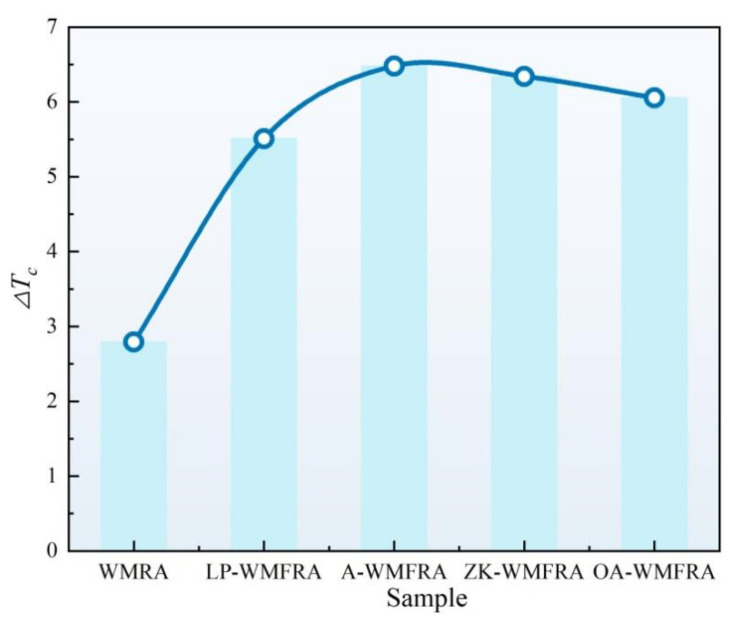
The results of ∆*T_c_* for WMFRA.

According to Figure 11, the S of WMFRA with a flame retardant was higher than that of WMRA, indicating that the flame retardant reduced the low-temperature flexibility of asphalt. At −24 °C, the S of all WMFRAs failed to meet the specification. However, it would be difficult to determine the effect of flame retardants on the low-temperature performance of WMRA with a single S or m value, so further evaluation with the help of ∆*T_c_* value was still needed. As can be seen in Figure 12, the ∆*T_c_* value of all asphalts were greater than zero, which suggested that the low-temperature behavior of WMRA and WMFRAs was mainly controlled by S(t) [45,46]. Li et al. [47] suggested limiting ∆*T_c_* to −5 °C to prevent non-load related cracking due to poor relaxation properties. As can be seen from Figure 11, all asphalt binders satisfied the minimum ∆*T_c_* criterion.

### 3.5. Fatigue Properties

**Figure 13:**
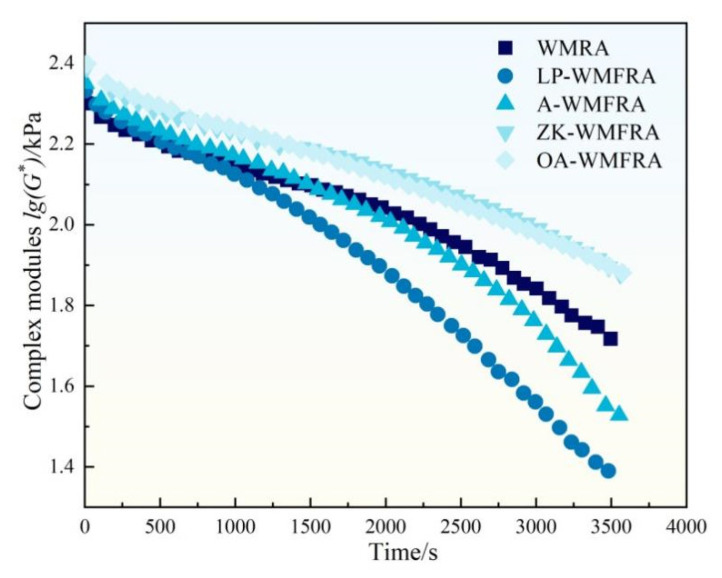
The complex modules of WMFRA in time sweep test.

The results of the time sweep are shown in Figure 13 and Table 8. The G* of LP-WMFRA decreased to below 50% at 1881 s after loading, showing a decrease of 27.2% compared with that of WMRA. This indicated that the incorporation of LP deteriorated the recovery capacity of WMFRA after load-induced deformation [48,49]. In contrast, the curves of OA-WMFRA and ZK-WMFRA almost overlapped and were higher than that of WMRA. The *N_f50_* of A-WMFRA and LP-WMFRA were smaller than that of WMRA, and the incorporation of ATH and LP caused WMFRA to reach the failure criterion 669.7 s and 958.9 s earlier, respectively. On the contrary, ZK increased the *N_f50_* of WMFRA by 3.2%, indicating that it could improve the fatigue performance of WMFRA. This was due to the fact that, on the one hand, the flame retardant can form a cross-linking structure with asphalt molecules to enhance the rigidity of the molecular chain but also capture the oxidizing radicals to terminate the chain aging reaction to reduce molecular fracture and brittle deterioration of asphalt under repeated loading. On the other hand, the flame retardant can form chemical bonds with the aggregate surface or fill the microscopic voids to avoid fatigue cracks caused by interfacial stripping [50]. The *N_f50_* of OA-WMFRA was approximately equal to that of WMFRA, suggesting that their fatigue performances were comparable.

**Table 8:** The *N_f50_* of WMFRA.

Sample	*N_f50_* (s)
WMRA	2765.4
LP-WMFRA	1806.5
ZK-WMFRA	2857.2
A-WMFRA	2095.7
OA-WMFRA	2790.5

## 4. Conclusions

In this paper, flame-retardant and rheological properties of warm mix flame-retardant asphalt (WMFRA) were characterized with different types of flame retardants. The main conclusions were as follows:

(1) The S-type warm mix agent significantly improved the fluidity of asphalt by reducing its viscosity by 46.7% at 135 °C but slightly reduced the low-temperature cracking resistance. However, R-type warm mix agent had the ability to enhance the low-temperature performance of asphalt but decreased the high-temperature stability, and the viscosity was reduced by a relatively small amount of 15.1% at 135 °C.
(2) The OA flame retardant composed of aluminum hydroxide (ATH) and organic modified montmorillonite (OMMT) had the best flame-retardant effect on WMFRA, and when OA dosage reached 15%, due to the synergistic effect of ATH’s heat absorption and water release and OMMT’s oxygen-blocking effect, the oxygen index was increased to 24%, which was significantly more than the application requirement of 23%.
(3) The OA flame retardant improved the high-temperature performance of WMFRA most significantly, with its complex modulus and rutting factor significantly higher than those of other WMFRAS. In addition, the incorporation of flame retardants generally reduced the temperature sensitivity of WMFRA, with the ZK flame retardant showing the most prominent improvement in temperature sensitivity by 12.6%.
(4) LP (limestone power) and ATH could enhance the stress relaxation of WMFRA, whereas ZK (zinc borate) and OA could be detrimental to the low-temperature performance of WMFRA, and all flame retardants would lead to a decrease in the low-temperature flexibility of WMFRA to some extent. According to the results of ΔT_c_, the low-temperature behavior of all WMFRAs was mainly controlled by S(t).
(5) LP and ATH dramatically lowered the fatigue life of WMFRA, with *N_f50_* reduced to 958.9 s and 669.7 s, respectively, while ZK improved fatigue life by 3.2% through the formation of a cross-linked structure. The *N_f50_* of OA-WMFRA was comparable to that of WMRA, but its complex modulus declined more slowly, suggesting that the flame retardant may have inhibited deformation of the asphalt while increasing rigidity recovery ability.

## Figures and Tables

**Table 1:** The main technical indicators of matrix asphalt.

Item		Index	Measured Value
25 °C Penetration (0.1 mm)		60~80	64
Softening point (°C)		≮46.0	47.5
15 °C Ductility (cm)		≮100	>100
Kinetic viscosity 60 °C (Pa·s)		≮180	219
Flash point (°C)		≮260	282
Wax content (%)		≯2.2	1.4
TFOT	Loss of mass (%)	≯0.8	0.03
	Penetration ratio (%)	≮65	67.9
	Residual ductility (10 °C, cm)	≮6.0	7.6

**Table 2:** The main technical index of S-type warm mixing agent.

Indicators	Density (25 °C, g/cm^3^)	Appearances	Melting Point (°C)	Flash Point (°C)
Measured value	0.89	Solid at normal temperature	>90	283

**Table 3:** The main technical index of R-type warm mixing agent.

Indicators	Density (25 °C, kg/L)	Appearances	Moisture Content	Flash Point (°C)
Measured value	0.93	Black green liquid at normal temperature	0.36	230

**Table 4:** The main technical index of ATH.

Item	Index	Measured Value
Al(OH)_3_ (%)	≮99.5	99.61
Whiteness (%)	≮96.5	96.7
Al_2_O_3_ (%)	≮64	66
SiO_2_ (%)	≯0.025	0.018
Na_2_O (%)	≯0.32	0.29
Fe_2_O_3_ (%)	≯0.02	0.012
pH	7.5~8.5	7.6
Mean particle size (μm)	28 ± 0.2	28.1
Absorption (mL/20 g)	≯6.2	5.8

**Table 5:** The main technical index of ZK.

Item	Measured Value
Content (%)	95.10
Free water (105 °C, %)	0.38
Particle size	fine type
Apparent color	white gray powder

**Table 6:** The main technical index of OMMT.

Item	Measured Value
Particle size (mesh)	325
Density (g/cm^3^)	1.8
XRD (d_001_/nm)	2.7
Ammonia content (%)	10.8

**Table 7:** The main technical index of LP.

Item		Index	Measured Value
Apparent relative density		>2.5	2.675
Particle size range (%)	<0.6 mm	100	100
	<0.15 mm	90~100	95.4
	<0.075 mm	70~100	78.7
Hydrophilicity coefficient		-	0.75
Plasticity index (%)		-	4.3
Heating stability		-	stable

## Data Availability

The original contributions presented in this study are included in the article.

## References

[1] Azadgoleh M.A., Mohammadi M.M., Ghodrati A., Sharifi S.S., Palizban S.M.M., Ahmadi A., Vahidi E., Ayar P. (2022). Characterization of contaminant leaching from asphalt pavements: A critical review of measurement methods, reclaimed asphalt pavement, porous asphalt, and waste-modified asphalt mixtures. Water Res..

[2] Jiang X., Zhu H.H., Yan Z.G., Zhang F.S., Huang X.Y., Leng Z., Yan C.Q., Hua N., Lu D., Zhang X.H. (2024). Fire-retarding asphalt pavement for urban road tunnels: A state-of-the-art review and beyond. Fire Technol..

[3] Leng C., Lu G.Y., Gao J.L., Liu P.F., Xie X.G., Wang D.W. (2019). Sustainable green pavement using bio-based polyurethane binder in tunnel. Materials.

[4] Feng S.Z., Kan D.Y., Zhou L., Liu X.L., Du C.Y., Mao W.X. (2025). Experimental study on effect of pavement background on obstacle visibility in LED lighting environment of road tunnel. Undergr. Space.

[5] Song K.P., Pan Y.T., Zhang J., Song P.A., He J.Y., Wang D.Y., Yang R.J. (2023). Metal–organic frameworks–based flame-retardant system for epoxy resin: A review and prospect. Chem. Eng. J..

[6] Patel R., Chaudhary M.L., Patel Y.N., Chaudhari K., Gupta R.K. (2025). Fire-Resistant Coatings: Advances in Flame-Retardant Technologies, Sustainable Approaches, and Industrial Implementation. Polymers.

[7] Xiao F.P., Guo R., Wang J.G. (2019). Flame retardant and its influence on the performance of asphalt—A review. Constr. Build. Mater..

[8] Qiu J.L., Yang T., Wang X.L., Wang L.X., Zhang G.L. (2019). Review of the flame retardancy on highway tunnel asphalt pavement. Constr. Build. Mater..

[9] Liu H., Zhang Z.P., Wang Z.F., Sun J., Wei Y.M., Zhang D.L. (2023). Preparation and properties of flame-retardant asphalt containing polyurethane and eco-friendly flame retardants. Constr. Build. Mater..

[10] Tian B., Li R., Wang D. (2012). Preparation of flame-retardant asphalt for tunnels. Adv. Mater. Res..

[11] Li W.B., Zheng M.L., Zhang L.W., Peng P., Yang J.P. (2024). Development and performance evaluation of composite flame retardant based on the thermodynamic properties of asphalt components. Constr. Build. Mater..

[12] Tan Y.W., Xie J.G., Wang Z.Q., Li K., He Z.Y. (2023). Effect of halloysite nanotubes (HNTs) and organic montmorillonite (OMMT) on the performance and mechanism of flame retardant-modified asphalt. J. Nanoparticle Res..

[13] Yang M., Yuan B.H. (2025). Modification of Aluminum Hydroxide by Ball Milling: A Feasible Method to Obtain High-Efficiency Flame Retardants for Production of High-Performance EVA Composites. Materials.

[14] Liu C., Zong R.W., Chen H.Y., Wang J.L., Wu C.P. (2019). Comparative study of toxicity for thermoplastic polyurethane and its flame-retardant composites. J. Thermoplast. Compos. Mater..

[15] Wang Z., Feng Z.G., Cai F.J., Zheng K.X., Li X.J. (2024). Flame retardancy and rheological properties of warm-mix rubber asphalt binder containing various flame retardants. J. Mater. Civ. Eng..

[16] Pan R. (2024). Fatigue Performance Evaluation of Warm-Mixed Rubber Asphalt Mixture for Stress Absorption Layer in Cold Area. Buildings.

[17] Choudhary R., Julaganti A., Kumar A., Ugale D.A. (2018). Application of WMA technology to bituminous base course mixes. Balt. J. Road Bridge Eng..

[18] Gong C.H., Jiang J.W., Liu T.C., Zhang R.H., Xu D. (2025). Compaction optimization of surfactant-tailored basalt-fiber WMA by improved lubrication and micro-structure formation. Constr. Build. Mater..

[19] Topal A., Sengoz B., Kok B.V., Yilmaz M., Dokandari P.A., Oner J., Kaya D. (2014). Evaluation of mixture characteristics of warm mix asphalt involving natural and synthetic zeolite additives. Constr. Build. Mater..

[20] Aljbouri R.Q., Albayati A.H. (2023). Investigating the effect of nanomaterials on the Marshall properties and durability of warm mix asphalt. Cogent Eng..

[21] Han Y.F., Duan P.P., Yu F., Yang A.Y., Zeng S.H., Chen P.P., Xu Y., Nie W.Y., Min Z.H., Zhou Y.F. (2024). Tuning high-and low-temperature rheological properties of warm-mixing asphalt composites by functionalized waxes. Mater. Today Commun..

[22] Gao Z.W., Fu H., Chen Q., Cao Y.S. (2020). Rheological properties and viscosity reduction mechanism of SBS warm-mix modified asphalt. Pet. Sci. Technol..

[23] Dong F.Q., Yu X., Liang X.M., Liu S.J., Ding G.Y., Xu B. The Influence of Foaming Water Content on the Aging Characteristic of Foamed Warm-Mix Asphalt. Proceedings of the Transportation Research Congress 2016: Innovations in Transportation Research Infrastructure.

[24] Huang X.Y., Ban Y.Y., Du L., Wang L. (2025). Molecular-Scale Study of the Compatibility of Warm-Mix Agents, Rubber, and Asphalt in Warm-Mix Rubber Asphalt. J. Mater. Civ. Eng..

[25] Chen B., Dong F.Q., Yu X., Ren S.S., Zheng C.J. (2022). Chemo-rheological characterization of aging behaviors of warm-mix high-viscosity modified asphalt. J. Mater. Civ. Eng..

[26] Tong B., Song X.Y., Shen J.A., Jiang T., Chen J.F., Niu J.F. (2022). Effect of Sasobit warm mix on micro properties of asphalt with different degrees of regeneration. Front. Mater..

[27] Zhang J.Z., Li P.Z., Sun C.J., Liang M., Li Y.Y., Yao Z.Y., Zhang X.M. (2019). Effects of composite warm mix additive (CAR) on the physical and rheological performance of bitumen and the pavement performance of its concrete. Materials.

[28] Wang J.J., Li H.Q., Chen X.G., Peng C. (2025). Influence of different types of warm mix agents on the viscosity reduction effect of Karamay modified asphalt. China Highw..

[29] Qin W., Yang J.Z., Ma F., Yang Y.F. (2025). Research on the preparation and performance of warm-mixed high-viscosity modified asphalt in alpine region. Appl. Chem. Eng..

[30] Tang N., Wang H.Y., Fu D., Wu L.F., Wang Q. (2020). Research progress on warm mixing technology of asphalt mixture. China Mater. Prog..

[31] He L., He Z.Y., Ling T.Q., Ma T., Huang X.M. (2015). Research on construction workability of asphalt-rubber mixture with warm mix additives. Funct. Mater..

[32] He S. (2021). Research on the Application of Warm Mixing Technology for Modified Asphalt Pavement in Expressway Tunnels. Master’s Thesis.

[33] Wu B. (2015). Research on the Flame Retardant System and Road Performance of Composite Hydroxide Asphalt. Master’s Thesis.

[34] Wu H.S., Shen A.Q., He Z.M., Deng S.Y., Wang K., Li Y. (2023). Mechanism of a warm-mix agent and its effects on the rheological properties and thermal stability of aluminum hydroxide and organic montmorillonite composite flame-retardant asphalt. J. Mater. Civ. Eng..

[35] Liu S.J., Wang H.M., Zeng L.H., Jiao X.D. (2024). Rheological Properties and Micromechanism of Warm-Mix Flame-Retardant Asphalt. J. Mater. Civ. Eng..

[36] Xu F., Li Zy Liu Y.Z., Xie H.J., Long Z.W., Dai B.T., Yang H., Zhu C.Z., You L.Y., Jin D.Z. (2025). Microscopic morphology and adhesion performance of SBS/OMMT modified asphalt under chloride salt erosion. Constr. Build. Mater..

[37] Ghasemirad A., Bala N., Hashemian L. (2020). High-temperature performance evaluation of asphaltenes-modified asphalt binders. Molecules.

[38] Zhang X.R., Han C., Yang J., Xu X.Q., Zhang F. (2021). Evaluating the rheological properties of high-modulus asphalt binders modified with rubber polymer composite modifier. Materials.

[39] Wang T., Jiang W., Ruan C., Xiao J.J., Yuan D.D., Wu W.J., Xing C.W. (2023). The rheological properties of high-viscosity modified reclaimed asphalt binder at multiple application temperatures. Constr. Build. Mater..

[40] Niu Y.H., Wang X.Y., Burmistrov I., Niu D.Y. (2024). Rheological properties and 3D printability of SBS/CR-modified asphalt binder with C9 petroleum resin for crack filling. Front. Mater..

[41] Jia M., Zhang Z.P., Liu H.T., Peng B., Zhang H.L., Lv W.J., Zhang Q., Mao Z.Y. (2019). The synergistic effect of organic montmorillonite and thermoplastic polyurethane on properties of asphalt binder. Constr. Build. Mater..

[42] Mirzaiyan D., Ameri M., Amini A., Sabouri M., Norouzi A. (2019). Evaluation of the performance and temperature susceptibility of gilsonite-and SBS-modified asphalt binders. Constr. Build. Mater..

[43] Arshadi M., Taherkhani H. (2024). Investigating the properties of asphalt binder modified by high-and low-density polyethylene polymer and nano-silica. Road Mater. Pavement Des..

[44] Sun M.K., Guo Q., He Z.Y., Yuan L., Sun D.X., Fang K., Wei D.B. (2025). Multi-objective optimization and performance investigation of surface modification synergistic warm-mixed flame retardant asphalt. Constr. Build. Mater..

[45] Chen X.B., Wang J.T., Zhang X.R., Liu H., Tong J.H., Zhao R.L. (2020). Evaluating the Physical and Rheological Properties of Rejuvenated Styrene-Butadiene-Styrene-Modified Asphalt Binders. Adv. Mater. Sci. Eng..

[46] Ma F., Zhu C.X., Fu Z., Li C., Hou Y.J., Jiang X.Y., Wu M. (2023). Analysis of rheological behavior and anti-aging properties of SBS modified asphalt incorporating UV absorbent and naphthenic oil (NPO). Constr. Build. Mater..

[47] Li X., Gibson N., Andriescu A., Arnold T.S. (2017). Performance evaluation of REOB-modified asphalt binders and mixtures. Road Mater. Pavement Des..

[48] Zhang Z., Han S., Guo H.C., Han X., Wu C. (2021). Fatigue performance evaluation of recycled asphalt fine aggregate matrix based on dynamic shear rheometer test. Constr. Build. Mater..

[49] Xiong L., Liu K., Kadhim H.A., Niu D.Y., Gao Y.M., Liu X.Y. (2025). Comparative analysis of the fatigue characterisation of natural rock asphalt/SBS composite modified asphalt binders using time sweep test and linear amplitude sweep test. Constr. Build. Mater..

[50] Tang N.P., Yu H.L., Ren B., Li R., Yi X.Y., Zhu H.Z. (2025). Effect of lanthanum oxide and magnesium hydroxide on flame retardancy, rheological properties, and aging resistance of SBS modified asphalt. Constr. Build. Mater..

